# Plasticity of Cu nanoparticles: Dislocation-dendrite-induced strain hardening and a limit for displacive plasticity

**DOI:** 10.3762/bjnano.4.17

**Published:** 2013-03-07

**Authors:** Antti Tolvanen, Karsten Albe

**Affiliations:** 1 Technische Universität Darmstadt, Institut für Materialwissenschaft, Fachgebiet Materialmodellierung, Petersenstr. 32, 64287 Darmstadt, Germany; 2 Department of Physics, FIN-0014 University of Helsinki, PO Box 43, Helsinki, Finland

**Keywords:** dislocation interactions, mechanical properties, molecular dynamics, nanoparticle, simulation

## Abstract

The plastic behaviour of individual Cu crystallites under nanoextrusion is studied by molecular dynamics simulations. Single-crystal Cu fcc nanoparticles are embedded in a spherical force field mimicking the effect of a contracting carbon shell, inducing pressure on the system in the range of gigapascals. The material is extruded from a hole of 1.1–1.6 nm radius under athermal conditions. Simultaneous nucleation of partial dislocations at the extrusion orifice leads to the formation of dislocation dendrites in the particle causing strain hardening and high flow stress of the material. As the extrusion orifice radius is reduced below 1.3 Å we observe a transition from displacive plasticity to solid-state amorphisation.

## Introduction

In macroscopic metals, the plastic flow is carried by the continuous movement, multiplication, and entanglement of mobile dislocations. As system size decreases, the relative surface (nanoparticle) or interface area (nanograined material) increases, and nucleation or annihilation of dislocations at surfaces or interfaces becomes a dominant factor since conventional dislocation sources, such as Frank–Read sources, are suppressed. This is commonly cited as the reason for the high mechanical strength of nanoscale materials [1].

Nanoscale systems also exhibit modes of plasticity not encountered in their macroscopic counterparts. Nanowires, for example, tend to respond to high tensile strain rates by amorphisation [2] attributed to the kinetic energy of atoms exceeding the enthalpy of fusion [3]. Also, a near-surface nanodisturbance path, where, instead of conventional displacive plasticity, nanoscopic areas of plastic shear accommodate the stress, was reported for Ag nanowires at high stresses and zero temperatures [4]. Non-close-packed nanostructures have been reported to deform by phase-transitions to a higher density phase. A limit of displacive plasticity leading to a phase-transition path was reported for Si nanospheres [5] concluding that in ultrasmall structures, where dislocation activity is suppressed, this path should dominate. Also in the tensile testing of twinned fcc Fe nanowires, a phase-transition path was reported as the dislocation activity is suppressed by the dense twin boundaries [6]. These findings raise a question: could there also be a size limit for the displacive plasticity of fcc metals?

Even though individual metal nanocrystallites would seem the simplest possible system in which to study nanoscale plasticity, they have not been well studied. One reason for this has been the complicated methods required to experimentally probe these systems. In a recent development, Sun et al. [7] reported a method in which individual nanocrystals are embedded inside nano-onions and pressurised by the contraction of the graphitic shells under electron irradiation. The contraction stems from the remarkable self-healing of the hexagonal network of carbon atoms as in fullerenes, carbon nanotubes, and graphene [8–9].

These nano-onions contract by electron-irradiation-induced defect formation and can exert forces in the gigapascal range on the encapsulated system. A hole punctured during the pressurisation allows the material to flow out after a threshold pressure, depending on the material properties, is reached. In their pioneering work, Sun et al. [7] studied the extrusion of Ag nanoparticles experimentally and attributed the plastic flow to dislocation activity, based on a combination of simulation results on Pt showing traces of dislocation activity (stacking faults within the encapsulated material even though the extruded material was not crystalline) and thermodynamical arguments stating the insufficient speed of diffusion for vacancy-assisted creep in the experimental system. Yet, neither dislocation nucleation nor dislocation interactions were observed in the computational study.

In this paper, we study the plasticity of Cu nanocrystallites in nanoextrusion. We show that dislocation pileup leading to the formation of dislocation dendrites inside the particle leads to strain hardening and limits the plastic flow. This supports the observed dislocation accumulation in nanograined materials [10–11]. We report novel dislocation interactions activated by the high pressure and dislocation density, and low dislocation length in the nanoparticle. We also show that the dislocation activity becomes suppressed as the extrusion hole radius is reduced to 12 Å and the mode of plasticity is changed from displacive to surface amorphisation.

## Results and Discussion

**Figure 1:**
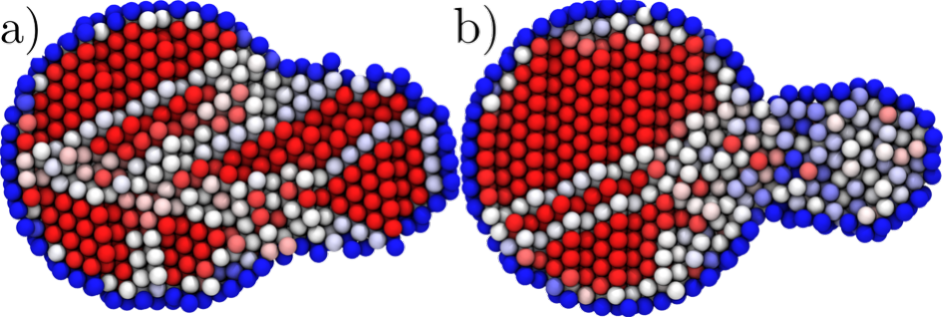
Two observed modes of plasticity. (a) Snapshot of the extrusion from a 15.6 Å orifice showing the displacive plasticity (b) The same from a 11.6 Å orifice showing the amorphous region about the orifice. Atoms coloured by the centrosymmetry parameter, from red (= 0, fcc) to blue (>25, surface).

Depending on the orifice radius, the mode of plasticity is either displacive or driven by surface amorphisation (see Figure 1 and Supporting Information File 1, and Supporting Information File 2). We start our analysis by presenting the details of the displacive case.

**Figure 2:**
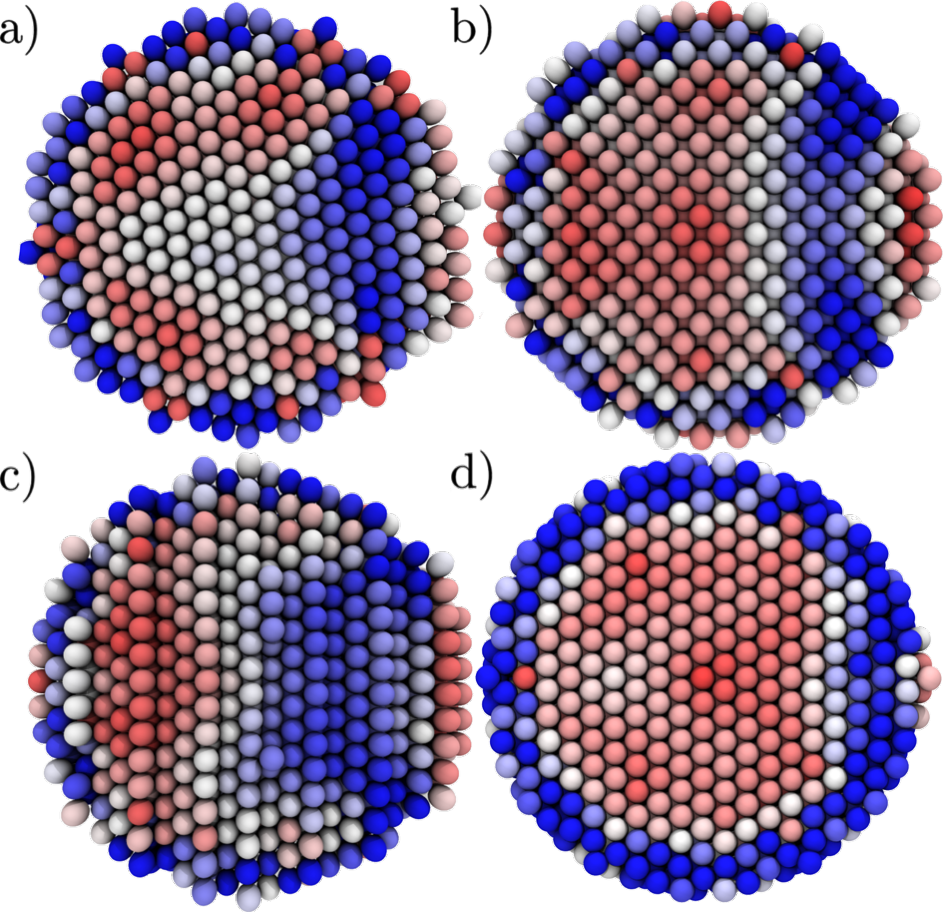
The maximum shear component of the atomic stress tensor expressed by the colouring of the atoms (colouring from red (= 0) to blue (>1 GPa)). (a–c) 15.6 Å orifice (displacive mode). (d) 11.6 Å orifice (surface amorphisation). (a) {111} planes oriented parallel to the extrusion direction. (b) {111} planes tilted with respect to the extrusion direction. (c) {111} planes oriented perpendicular to the extrusion direction. (d) {111} planes parallel (as in (a)) at the inset of surface amorphisation. To relieve the stress the systems have slightly rotated inside the force field.

The maximum shear component (the maximum eigenvalue of the atomic stress tensor) in the initial system and at the onset of plasticity for different orientations for a 15.6 Å orifice is illustrated in Figure 2a–Figure 2c. For all the orientations, larger than average values of maximum shear are roughly localised on inverted spherical caps joining the edge of the circular orifice.

The most extreme values of the atomic shear (parallel 2.4 MPa, orthogonal 2.3 MPa, and tilted 2.3 MPa) are almost the same and are located at the intersection of the orifice and the surface where the dislocations are nucleated. Before the onset of plasticity, the system goes through small rotations to accommodate the stresses changing slightly the initial lattice orientation.

Qualitatively, in the displacive regime, the deformation follows the same route in all our simulations. At the onset of plasticity, multiple dislocations nucleate simultaneously at the surface of the particle near the borders of the extrusion orifice (see Figure S1 in Supporting Information File 3 for illustration of this in different orientations of the system). These dislocations form complicated lock structures, named here dislocation dendrites, inside the particle that have to be broken for the plastic flow to continue. As the qualitative behaviour of the system is almost independent of the orientation, we concentrate on the case where the {111} planes are parallel with the extrusion direction (Figure 2a) remarking where the behaviour differentiates depending on the direction.

**Figure 3:**
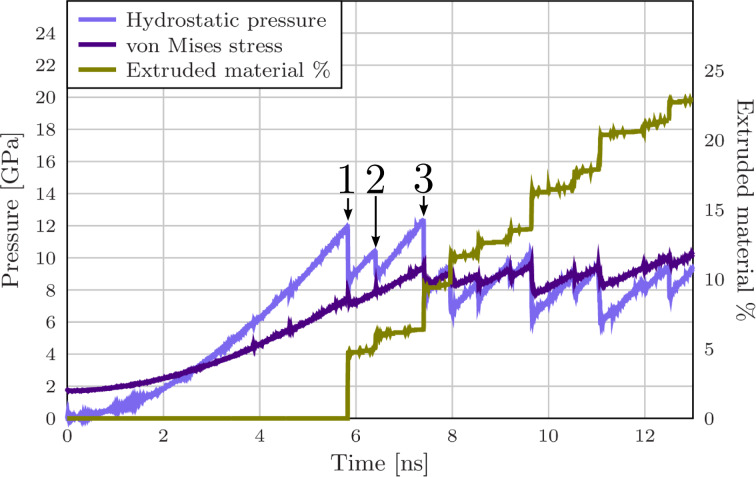
Extrusion from a 15 Å orifice. Hydrostatic pressure, von Mises stress and the amount of extruded material as a function of the simulation time. The nonzero initial value of the von Mises stress is due to the initial shear stresses at the faceted surface of the nanoparticle.

**Figure 4:**
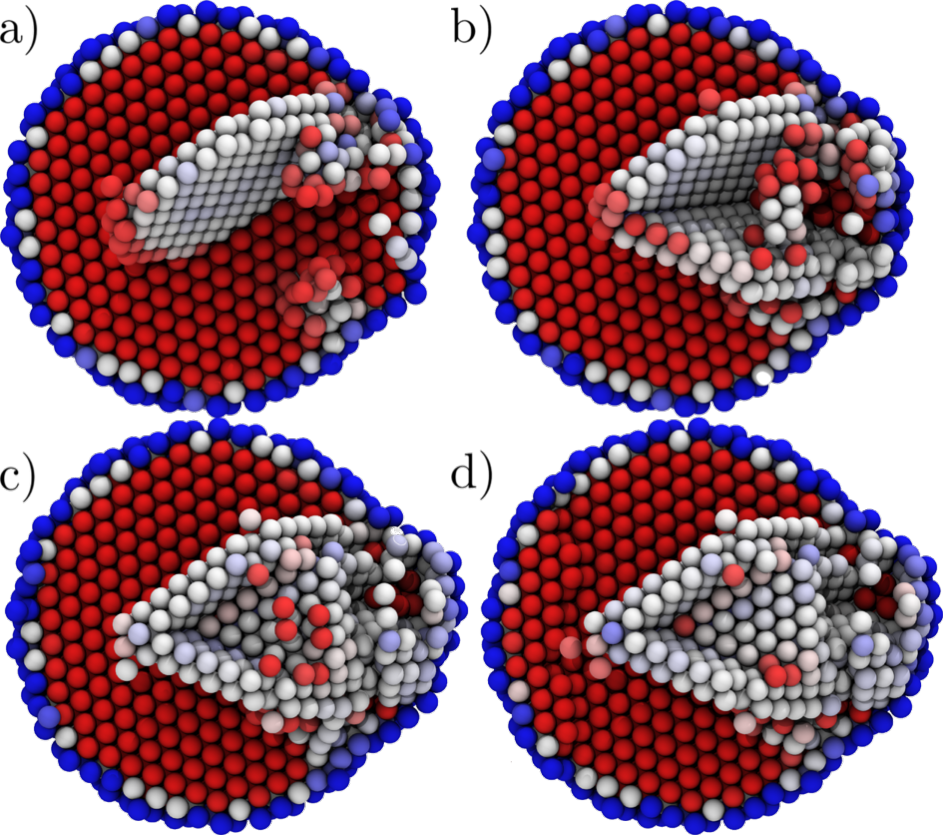
Extrusion from 15 Å orifice. (a) The nucleation of the first Shockley partial. (b) Two nonlocking sessile Shockley partials. (c) A stair rod is formed. (d) The dislocations are pinned to the particle surface through a <312> dislocation.

**Figure 5:**
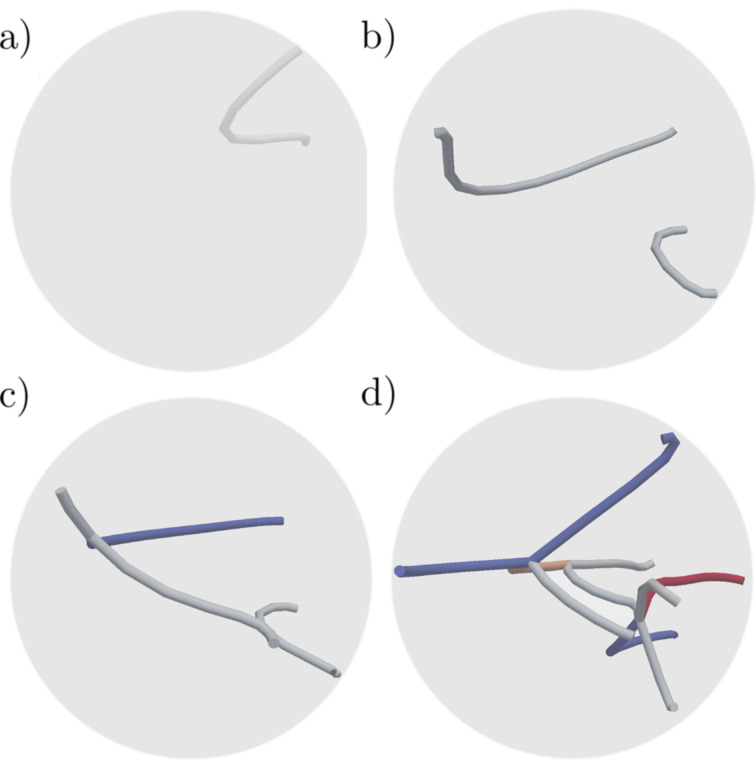
Dislocations interacting at the onset of plasticity. Colouring by the length of the Burgers vectors, red |
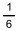
<312>|, grey |
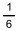
<112>|, and blue |
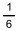
<110>|. (a–b) Shockley partials are nucleated 2.5 ps apart at the onset of plasticity. (c) Nucleation of a third dislocation leads to the formation of a stair-rod dislocation. (d) Nucleation of multiple dislocations leads to the formation of dislocation dendrite inside the particle blocking the dislocation nucleation and motion.

The stress–strain behaviour of this system during the extrusion process through an orifice of 15.6 Å radius is presented in Figure 3. Before the elastic regime, the system goes through a short plastic phase in which the faceted surface of the particle accommodates the spherical force field. The system yields at 11.9 GPa (2.9% strain, marked as 1 in Figure 3) when two Shockley partials are nucleated 2.5 ps apart (see Figure 4 and Figure 5). The first partial is soon met with a third partial nucleated at the opposite side of the orifice forming a 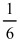
<011> stair-rod dislocation ((c) in Figure 4 and Figure 5). Such interactions of partial dislocations in Cu have been reported before as the obstacle for dislocation motion [12]. In the case of the nanoparticle under extreme stress, the stair-rod dislocations do not block the plastic flow, because the continuous nucleation–interaction–unzipping cycles make the Shockley partial-stair-rod systems unstable.

**Figure 6:**
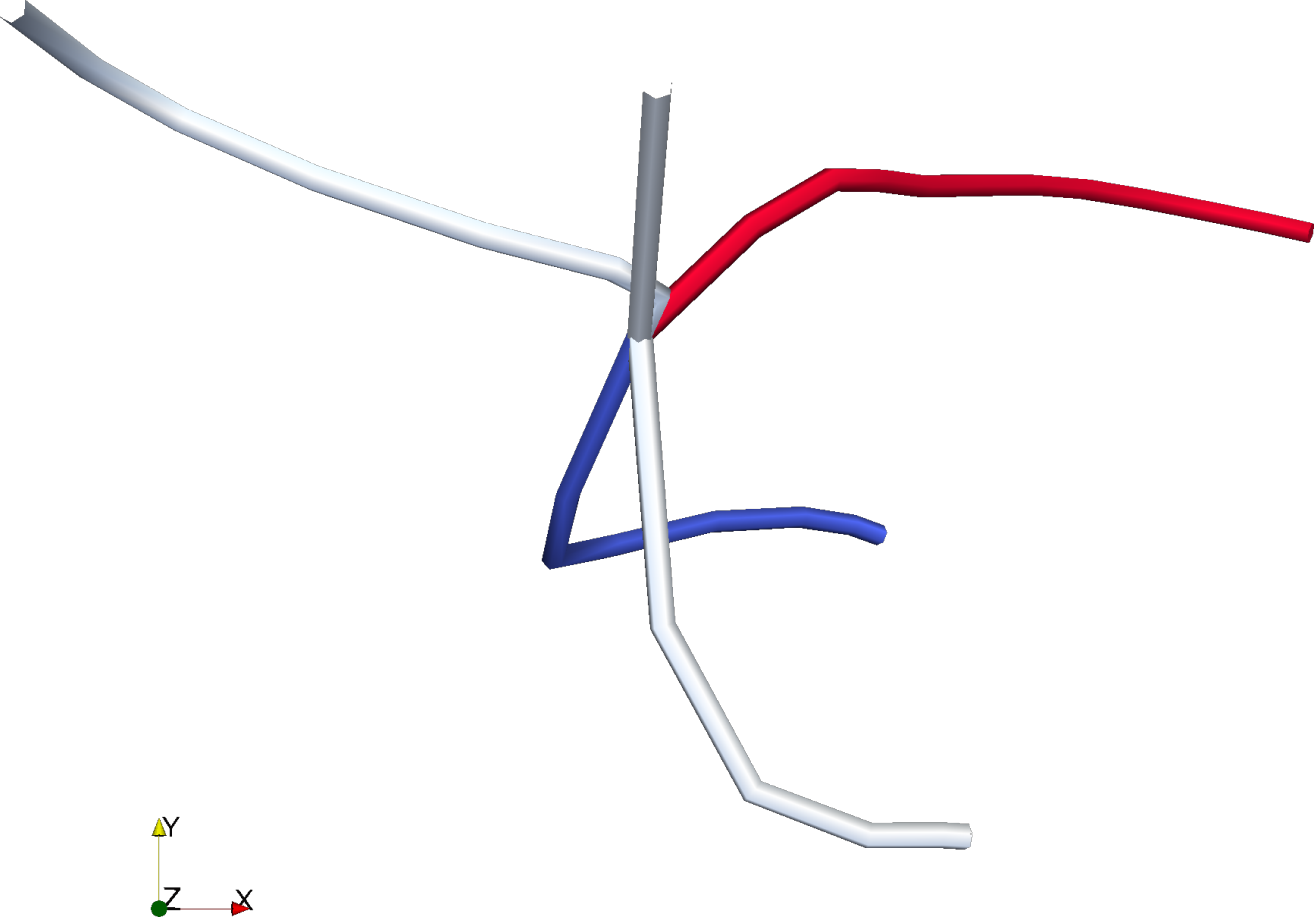
A dislocation multijunction blocking the dislocation mobility and nucleation in the system. Dislocations end at the right side of the figure on the particle surface, and the Shockley partial on the left connects to the other dislocations in the particle. Colouring by the length of the Burgers vectors, red |
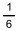
<312>|, grey |
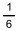
<112>|, and blue |
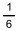
<110>|

Contrary to the common view of dislocation nucleation and annihilation at the surface of the grain, the dislocations can be stopped inside the particle also independently of any dislocation interaction, as shown in Figure 5b and Figure 5c, in which the second Shockley partial becomes sessile promptly after the nucleation. As the opposing surfaces of the particle are under extreme stress it would be unfavourable to accommodate the strain field associated with the partial approaching the constricted surface. This leads to accumulation of dislocations near the extrusion orifice and to dislocation interactions typically not found in bulk materials. An example of such a situation is presented in Figure 5d in which two stair-rod dislocations and a Shockley partial interact to form an unstable 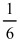
<130> dislocation (coloured coral in Figure 5d) (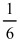
<
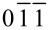
> + 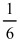
<
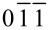
> + 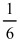
<
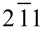
> = 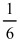
<
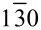
>) linking to another pair of partials (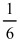
<
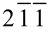
> + 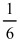
<
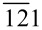
> = 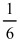
<
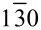
>). A typical feature is the locking of a dislocation multijunction to the particle surface through a 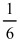
<312> dislocation (red in Figure 5d) here by a Shockley triple junction and a stair rod (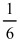
<
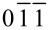
> + 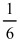
<
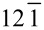
> + 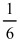
<
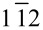
> + 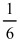
<
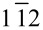
> = 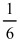
<
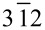
>). The system reaches a steady state 35 ps after the onset of plasticity, and the dislocation nucleation and movement becomes locked by a clawlike multijunction reaching the surface at the extrusion orifice (see Figure 6). Also in macroscopic materials, simpler dislocation multijunctions have been reported as a contribution to strain hardening [13].

**Figure 7:**
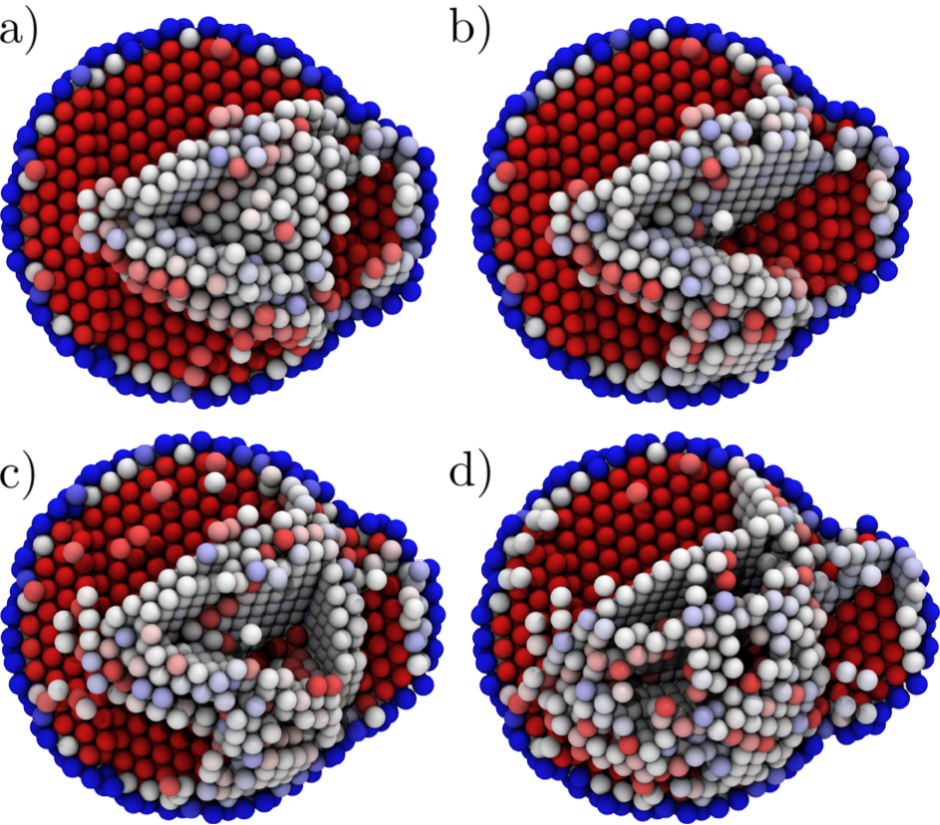
Atomic arrangement during the breaking of the dendrite. (a–b) The locking multijunction (see Figure 6) is broken as the 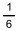
<112> partial is disassociated from the junction. (c–d) A partial nucleated at the particle surface (here lower right about the orifice) interacts with the partials in the dendrite pinned to the surface, resulting in a slip.

**Figure 8:**
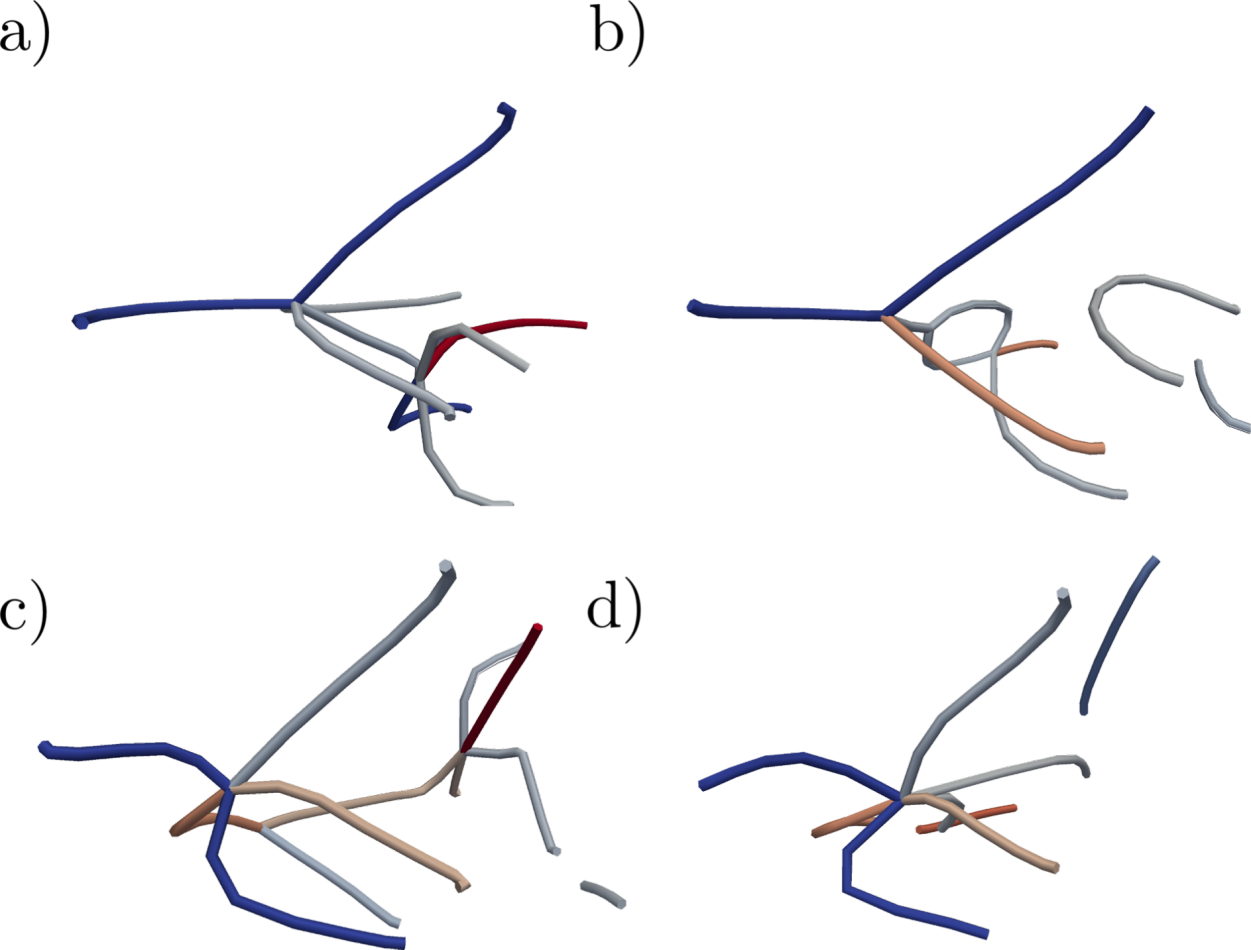
Dislocation interacting to break the dendrite. (a–b) The locking multijunction (see Figure 6) is broken as the 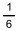
<112> partial is disassociated from the junction. (c–d) Partial nucleated at the particle surface (here lower right about the orifice) interacts with the partials in the dendrite pinned to the surface, resulting in a slip.

After locking the dislocations into dislocation dendrites, they have to be broken in order to continue the plastic flow. We observe two distinct breaking mechanisms: Disassociation of dislocations or interaction with new partials nucleated at the surface. Examples of these processes are presented in Figure 7 and Figure 8 (marked as points 2 and 3 in Figure 3). The disassociation mechanism we observe is not an unzipping process as discussed above in the case of the stair-rod dislocations. Here, a partial breaks of from the clawlike structure (Figure 7a and Figure 7b and Figure 8a and Figure 8b) and the remaining 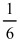

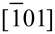
, 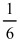

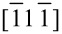
, 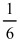
[121] and 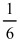
[312] are broken into two partials. Note that the system is on average constantly at the limit of the lattice instability of bulk Cu (≈10 GPa), allowing such violent processes. The nucleation–interaction mechanism is initiated, if in spite of the back stresses of the dislocations locked in the dendrites inside the particle, partials are nucleated at the surface. As an example, in Figure 7c and Figure 7d and Figure 8c and Figure 8d a partial from the surface immediately meets the sessile partials in the dendrite about the orifice, resulting in a slip and a residual stair rod.

**Figure 9:**
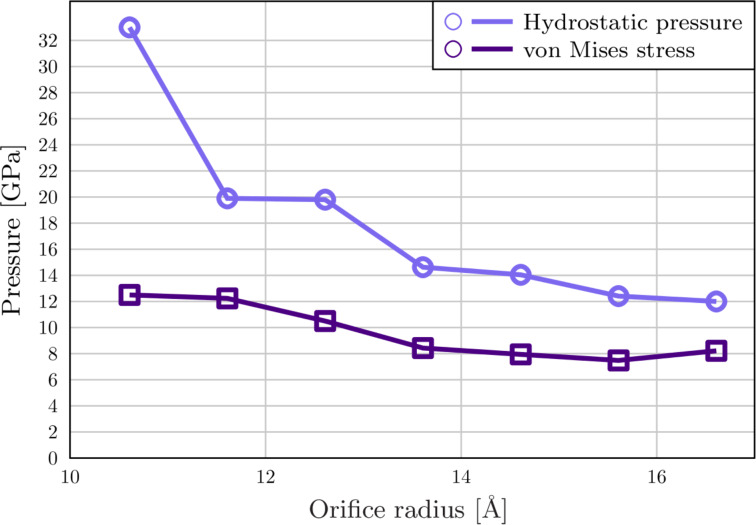
Hydrostatic pressure and von Mises stress at the onset of plasticity versus extrusion orifice radius. Lines are guides for the eye.

**Figure 10:**
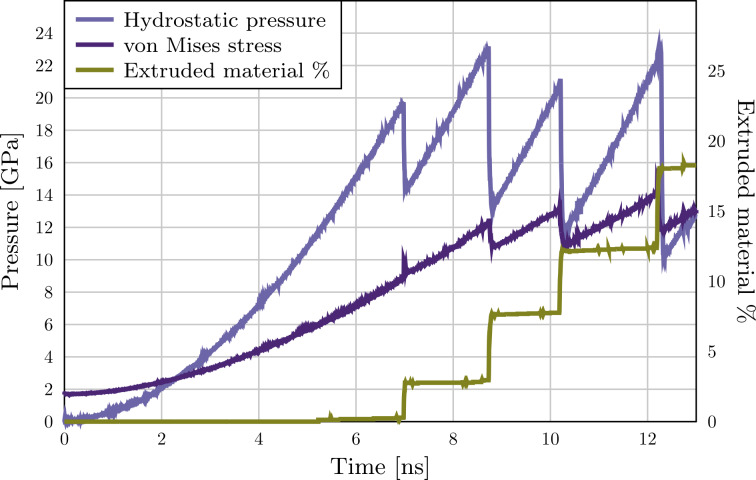
Extrusion from a 11 Å orifice. Hydrostatic pressure, von Mises stress and the amount of extruded material as a function of the simulation time. The nonzero initial value of the von Mises stress is due to the initial shear stresses at the faceted surface of the nanoparticle.

When the orifice size is reduced the maximum stress at the onset of plasticity is increased (see Figure 9) but the qualitative behaviour of the system remains the same down to an orifice radius of 12.6 Å. At this point, we observe that the displacive mode of plasticity becomes unfavourable in favour of a surface-amorphisation-induced plastic flow. As an example, we present the results for a parallel system with 11.6 Å orifice radius, see Figure 10 and Figure 1. Here at the onset of plasticity the surface of the particle breaks down leading to a burst of material out of the orifice. Von Mises stress at the onset of plasticity exceeds 1/5 *G* ≈ 10 GPa, where *G* = 46 GPa is the shear strength of Cu, suggesting a stress regime above the ideal shear strength, even locally at the orifice.

**Figure 11:**
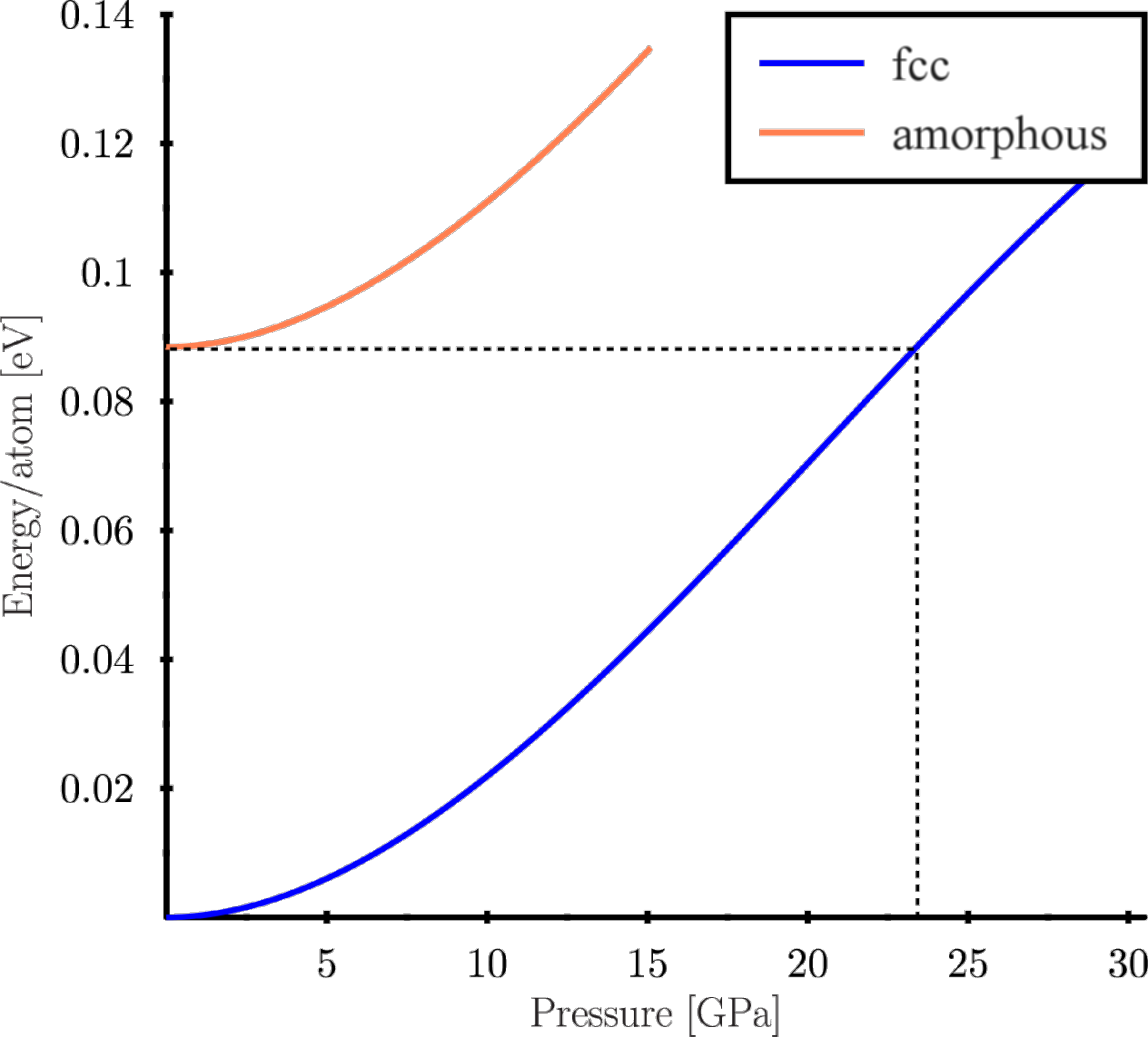
Energy per atom with respect to the fcc phase at zero pressure for fcc and amorphous Cu. Energy of the fcc phase coincides with the amorphous phase at 23.5 GPa.

An amorphous region is observed at the orifice throughout the whole simulation, and dislocations carry the plasticity only further inside the particle (see Supporting Information File 2). Such behaviour is observed at all radii below 12.6 Å. Why is the surface amorphisation favoured over displacive plasticity? In the fcc phase, the material is at the maximum possible density and amorphisation cannot release the pressure by contraction of the atomic volume. However, in the observed surface phase transition the amorphous phase is effectively at zero pressure, and beyond the pressure limit of ca. 20 GPa the energies of the pressurised fcc phase and the zero-pressure amorphous phase coincide and the system does not lose energy when transforming to the higher-energy phase, see Figure 11.

It is important to note, that even though the amorphous region shows liquid-like flow, the system temperature stays at about 0 K and the atoms in the amorphous region stay static between the bursts of the material. Thus this process is distinct from the high-strain-rate/high-momentum driven amorphisation observed in nanowires [2], where the threshold strain rates for amorphisation are more than three orders of magnitude higher than in this study. Still, it has to be noted, that the atomic pressures as such are not well-defined locally and can give only qualitative reasons.

Regarding the suppression of the dislocation activity, the most obvious limit would be the stress required to bend the dislocation pinned at the extrusion orifice, as this stress

[1]
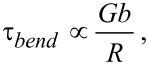


where *R* is the radius of curvature of the dislocation bending. However, as for Cu, *G* = 46 GPa, *b* = 1.48 Å and *R* = 10–16 Å. This leads to stresses in the range of 6.8–4.3 GPa, which the von Mises stress of the system exceeds in all the cases. Thus, such simple approximations cannot capture even the qualitative differences of the observed modes of plasticity. The only apparent difference, in addition to the hydrostatic pressure, is the initial stress distribution as seen in Figure 2d. At the lower orifice radii the shear stress is localised at the particle surface, and thus, even if dislocations attempting to accommodate the loading to the system would be nucleated, there would not be a shear to drive these inside the particle. Moreover, as the hydrostatic pressure of the system increases it becomes increasingly difficult for the dislocations nucleated at the surface to penetrate the material.

## Conclusion

In summary, we have used molecular dynamics simulations to show how the formation of dislocation dendrites consisting of multiple different types of dislocations leads to strong strain hardening of individual Cu nanocrystallites. We also report a variety of dislocation interactions, not observed in the deformation of macroscopic metals, taking place during the plastic flow from the nanoparticle. We suggest a high pressure limit for displacive plasticity at which the surface amorphisation of the particle becomes the more favourable mode of plasticity. Our computational study further elucidates how the nanoscale processes differ from the familiar macroscopic counterparts and motivates more studies to understand the possible limits of displacive plasticity.

## Methods

Molecular dynamics (MD) simulations were performed by using a modified version of the LAMMPS [14] simulation package. Interatomic interactions of Cu atoms were modelled by an EAM type inter atomic potential developed by Mishin et al. [15], which gives the correct generalised stacking fault energies [16]. In order to restrict the system to the regime of purely displacive plasticity, the system temperatures were kept close to 0 K by a Berendsen thermostat [17].

The nanoparticles were encapsulated inside a external repulsive spherical force field with a circular orifice interacting with the Cu atoms with a repulsive Lennard-Jones type potential. As the carbon atoms in a graphitic shell interact very weakly with the metal particle in equilibrium [18], and since during the contraction the interaction is repulsive, the exact functional form of this interaction is irrelevant, and such a simple model captures the essence of the process of a contracting carbon shell. Spherical nanoparticles were formed by cutting a sphere of radius 21.58 Å, with 3892 atoms, from an fcc Cu bulk. After cutting, the particle was annealed at 800 K for 500 ps and slowly cooled down to 0 K. Three different orientations aligning the {111} planes of the particle parallel, orthogonal, and tilted with respect to the extrusion direction, were chosen (referred to below as parallel, orthogonal and tilted).

The system was strained by reducing the radius of the external force field in 0.005 Å steps every 25 ps while keeping the radius and the position of the orifice constant. The radius of the extrusion orifice was varied from 16.6 to 10.6, which was found to capture the orifice-dependent changes in the plasticity. As the initial radius of the force field was 24.6 Å, this contraction rate corresponds to an initial strain rate of 8.1 × 10^−6^ ps^−1^ increasing to 8.5 × 10^−6^ ps^−1^ at the onset of plasticity. A constant contraction rate was selected over a constant strain rate, because it relates more accurately with the experimental setup modelled. A small time step of 0.5 fs was used to capture accurately the physics of the extremely fast processes taking place in the high-pressure nanosystem.

Atomic stresses and the pressure of the system were calculated from the atomic virials assuming constant atomic volumes, and the atomic stress tensors were diagonalised by using the Jacobi rotation method [19] for the analysis of shear components. With the multitude of possible dislocation interactions in a nanoscale system with a high dislocation density, it becomes cumbersome, or impossible, to identify the dislocations by using atomic energies, common neighbour analysis, or centrosymmetry parameters, which are often efficient and reliable in the studies of macroscopic systems in large-scale simulations. To overcome this problem, we employed a recent dislocation-detection algorithm developed by Stukowski et al. [20] and the related tool [21]. We found this method to be reliable and robust, even with the complicated surface effects and dense dislocation networks of our studied systems.

## Supporting Information

The Supporting Information files show the evolution of the nanoparticle during extrusion from a 15 Å orifice showing displacive plasticity; evolution of the nanoparticle during extrusion from a 11 Å orifice showing surface amorphisation; and simultaneous dislocation nucleation at the onset of plasticity in different orientations of the nanoparticle during extrusion from a 15 Å orifice.

File 1 Extrusion from a 15 Å orifice

File 2 Extrusion from a 11 Å orifice

File 3 Simultaneous nucleation in different orientations of the system
