# Supplementary material for: Plasticity of Cu nanoparticles: Dislocation-dendrite-induced strain hardening and a limit for displacive plasticity
Source: Beilstein J Nanotechnol. 2013 Mar 7;4:173–9. doi: 10.3762/bjnano.4.17 (PMC3628289; doi:10.3762/bjnano.4.17)
Supplement: File 3 — Simultaneous nucleation in different orientations of the system [file Beilstein_J_Nanotechnol-04-173-s003.pdf]

Supporting Information

for

Plasticity of Cu nanoparticles: Dislocation dendrite induced strain hardening and a limit for displacive plasticity

Antti Tolvanen*[1,2] and Karsten Albe[1]

Address: [1]Technische Universität Darmstadt, Institut für Materialwissenschaft, Fachgebiet Materialmodellierung, Petersenstr. 32, 64287 Darmstadt, Germany and [2]Department of Physics, FIN-0014 University of Helsinki, PO Box 43, Helsinki, Finland.

Email: Antti Tolvanen* - tolvanen@mm.tu-darmstadt.de

* Corresponding author

Simultaneous nucleation in different orientations of the system

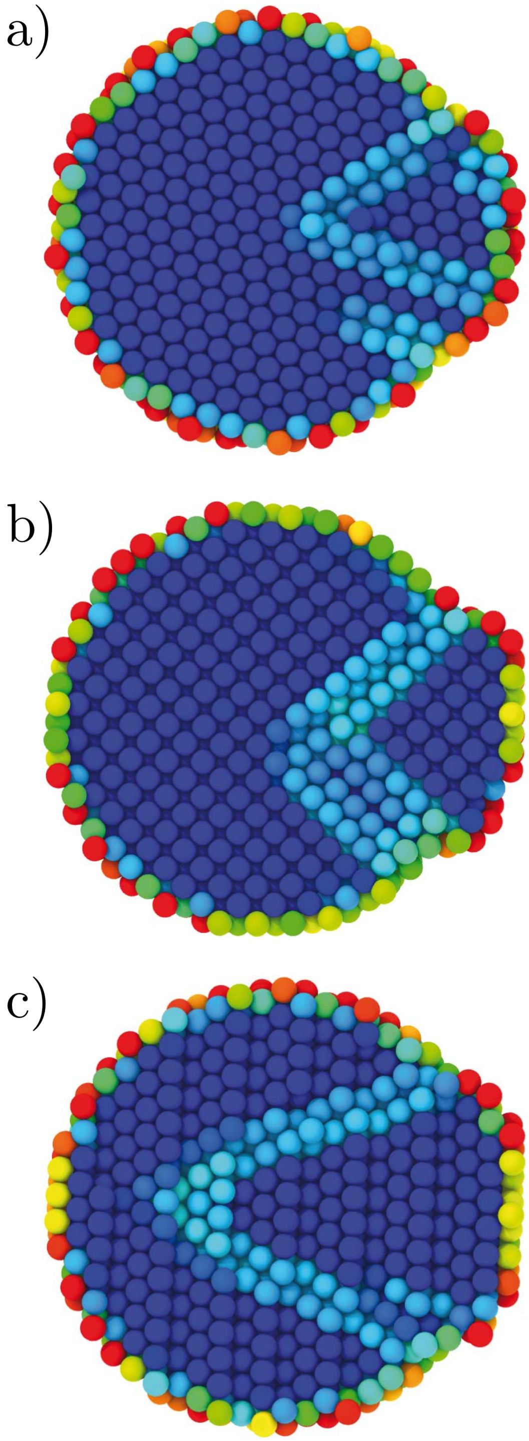

Figure S1: Simultaneous dislocation nucleation in different orientations of the system. a) Parallel, b) perpendicular, and c) tilted.
